# Xiaochaihu Decoction Treatment of Chicken Colibacillosis by Improving Pulmonary Inflammation and Systemic Inflammation

**DOI:** 10.3390/pathogens12010030

**Published:** 2022-12-25

**Authors:** Ke Song, Jiang Li, Yuerong Tan, Jiaying Yu, Miao Li, Siyang Shen, Luyuan Peng, Pengfei Yi, Bendong Fu

**Affiliations:** College of Veterinary Medicine, Jilin University, Changchun 130000, China

**Keywords:** traditional Chinese veterinary medicine, Xiaochaihu Decoction, chicken colibacillosis, avian pathogenic *Escherichia coli*, therapeutic effect

## Abstract

Chicken colibacillosis—the most common disease of poultry, is caused mainly by avian pathogenic *Escherichia coli* (APEC). It has a major impact on the poultry industry worldwide. The present study was conducted to investigate the therapeutic effects of Xiaochaihu Decoction (XCHD) supplementation on clinical manifestation, organ index, bacterial load in organ and inflammatory mediators in a chicken model challenged with APEC. The results showed that all doses of XCHD significantly elevated the survival rate of infected chickens. XCHD improved the clinical signs of infected chickens, reduced the organ index, reduced the bacterial load of organs, and inhibited the secretion of serum and pulmonary inflammatory factors IL-1β, IL-6 and TNF- α. Taken together, this study demonstrates that XCHD had protective effects on APEC-infected chickens. Its mechanism includes anti-inflammatory and antibacterial effects. These findings may contribute to the further study of the mechanism of the formula and the prevention or treatment of colibacillosis in poultry. The significance of this study is that it provides a certain theoretical basis for the replacement of antibiotics by XCHD.

## 1. Introduction

Avian pathogenic *Escherichia coli* (APEC) causes chicken colibacillosis and results in acute and mostly systemic infections in avians. Due to the age, resistance, pathogenicity and infection routes of infected chickens, Chicken Colibacillosis include many clinical signs and different disease types. There is a high incidence of diseases in chicken farming, which is responsible for high economic losses in the chicken industry [1,2]. Chicken colibacillosis is clinically characterized by a variety of disease types, including pericarditis, perihepatitis, peritonitis, air sac inflammation and septicemia [3].

At present, there are more than 60 types of *Escherichia coli* (*E. coli*) serotypes in China, including O1, O2, O35 and O78 [4]. Chickens of all ages are susceptible to colibacillosis when kept under poor hygienic conditions and when improperly fed. The usual incidence of chicken colibacillosis is 11% to 69% and the mortality rate is about 3.8% to 72%, which can reach 100% in severe cases [5].

Chicken colibacillosis is often secondary to other respiratory diseases in chickens. APEC is a symbiotic bacterium in the intestinal tract of animals and can become pathogenic through horizontal gene transfer and recombination with other pathogenic bacteria. As a result, the death rate of young birds has increased year by year [6].

The use of antibiotics can be effective in the treatment of chicken colibacillosis, but the misuse of antibiotics promotes resistance to APEC, leading to multidrug resistance and making the disease difficult to control [7]. However, the control of coliforms is complicated by the prohibition of antibiotics in feed. To cope with this, people choose to find natural alternatives with antimicrobial properties to maintain the level of production and product quality in the chicken industry [8,9]. Therefore, the search for a safe and effective antibiotic substitute is one of the key issues in the poultry industry and public health research.

Xiaochaihu Decoction (XCHD), a Chinese herbal formula, is composed of *Bupleurum*, *Scutellaria*, *Pinellia Ternata*, *Codonopsis Pilosula*, *Ginger*, *Jujube* with an optimal ratio that has attracted increasing attention as an alternative treatment and supplement. According to traditional Chinese medicine, the Xiao Chai Hu Tang formula can complement the healthy qi (a vital force or energy that can control the human body), dispel the unhealthy qi, and mediate qi and blood circulation in and around the liver and gallbladder [10]. 

XCHD is widely used in the treatment of hepatitis, liver fibrosis and airway inflammation [11,12,13]. *Bupleurum* extract can significantly reduce acute lung injury (ALI) induced by acetaminophen (APAP) and improve enteritis induced by dextran sulfate sodium salt (DSS) [14,15]. Intestinal pathological characteristics can be observed. The aqueous extract can protect the intestinal barrier by increasing the levels of intestinal barrier mucins (MUC1 and MUC2mRNA) and atresia band-1 (ZO-1) and Claudin-1 proteins [16,17]. Several traditional uses of *Scutellariae* have been proven by modern pharmacology, such as anti-inflammation and anti-infection. For instance, the water extracts of *Scutellaria* demonstrated anti-inflammatory activities on periodontitis through inhibiting the production of IL-1β, 6, 8 and TNF-α expression in gingival tissues and reduced the mineralization of 18 cementum [18,19,20]. Additionally, many compounds and extracts in genus *Scutellaria* showed great antibacterial activity in vitro [21]. Other ingredients in XCHD also have different functions such as reducing lung inflammation, improving immunity, the antioxidant effect, the hepatoprotective effect and so on [22,23,24]. Accordingingly, we hoped to provide a theoretical basis for further research on XCHD and its application in prevention of or therapy for chicken colibacillosis.

## 2. Materials and Methods

### 2.1. Bacterial Strain

APEC-O78 strain (CVCC1418) was obtained from the Chinese Veterinary Culture Collection Center (CVCC, Beijing, China) and store at −80 °C in the refrigerator The activated APEC-O78 single colony was picked into lysogeny broth (LB) medium (Qingdao Hope Bio Technogy Co., Ltd., Qingdao, China) and cultured on a shaker at 37 °C, 200 r/min for about 12 h, and the uninoculated LB medium was used as the control. SynergyTM HT multi-mode enzyme label Instrument (BioTek Instruments, Winooski, VT, USA) measured the corresponding OD600 value. The concentration of bacterial solution was made at 10^8^ cfu/mL.

### 2.2. Xiaochaihu Decoction Preparation

Xiaochaihu Decoction consists of *Bupleurum* 25 g, *Scutellaria* 10 g, *Pinellia Ternate* 10 g, *Codonopsis pilosula* 10 g, *Ginger* 10 g, *Jujube* 10 g (Tongrentang Pharmacy in Jilin Province, Changchun, China). After decoction, XCHD was concentrated to 1 g/mL.

### 2.3. Experimental Design and Animal Treatment

The experiments were conducted in a manner that avoided unnecessary discomfort to the animals using proper management and laboratory techniques. All the experimental procedures were approved by the Institutional Animal Care and Use Committee of Jilin University (Number of permits: KT201903080). In total, 120 one-day-old male hyline brown chickens (average BW = 50 g) were obtained from Changchun Academy of Agricultural Sciences. They were inspected upon arrival to ensure all chicks were free from any deformities and early signs of disease. The chicks were fed until they were 14 days old and were then randomly divided into 6 groups (20 chicks in each group). Until the end of the experiment, all groups were fed a basal diet without growth promoters and antibiotics. All diets were fed for ad libitum intake with free access to water.

Chickens in the positive control group and the XCHD treatment group were injected with APEC solution 0.4 mL through the pectoral muscle with a suspension concentration of 10^8^ cfu/mL [25]. The XCHD group were administered at doses of 2 g/kg, 1 g/kg, 0.5 g/kg (Table S1). The antibiotic group administered Ceftiofur sodium (Chengdu Prosperous Animal Pharmaceutical Co., Ltd., Chengdu, China). The treatment of chickens in each group is shown in Table 1.

### 2.4. Experimental Design and Sampling Collection

#### 2.4.1. Clinical Manifestation

After the establishment of the model, the clinical symptoms, mortality, and survival rate of the experimental animals were observed and recorded, and the dead chickens were examined at the same time.

#### 2.4.2. Body Weight

At the end of the experiment, all chickens were weighed for growth performance calculations.

#### 2.4.3. Organ Index

At the end of the observation, the chickens in each group were weighed, blood samples were collected from the jugular vein, anesthesia was performed, and the chicks were dissected. The pathological changes of chickens in each group were observed after dissection and the heart, liver, spleen, lung, kidney, and bursa of Fabricius were collected. The organ index was calculated after weighing, and the formula was as follows: organ index = organ weight (g)/body weight (kg) [26].

#### 2.4.4. Determination of Bacterial Load in Organs

Establishment of a standard curve. Dilute the prepared 10^8^ cfu/mL bacterial solution tenfold to 10^2^ cfu/mL, extract DNA from bacterial liquid according to the instructions of the TIANamp Bacteria DNA Kit (Tiangen Biochemical Technology Co., Ltd., Beijing, China), and establish a standard curve using the data detected by qPCR. Take the logarithm of the bacterial load in the reaction as the abscissa and the CT value in the PCR reaction as the ordinate, establish a standard curve and 16s rRNA gene of bacteria was amplified by qPCR. The forward primer was CACAATGGGCGAAAGCCTGA, and the reverse primer was GGCTGCTGGCACGTAGTTAG. 

Bacterial load in organ: After grinding the organ (heart, liver, spleen, lung, kidney and bursa of Fabricius, the sample weight of each organ is 0.1 g), the supernatant was obtained after 6000 rpm centrifugation for 10 min. The bacterial genome was extracted from tissues by TIANamp Bacteria DNA Kit, and the change of bacterial load was analyzed by qPCR. (Bestar sybrGreen RT Reagent Kit, ROCHE, Basel, Switzerland).

#### 2.4.5. Blood Biochemical Indicators

After the observation, blood was collected from the chickens of each group through the jugular vein, centrifuged at 3000 rpm for 10 min, serum was taken, and the aspartatealanine were tested according to the instructions of the test kit (Huili Biotechnology Co., Ltd., Changchun, China).

#### 2.4.6. Serum Inflammatory Factors

At the end of the observation period, blood samples were collected from the jugular vein, and 5 chickens in each group were randomly selected. The blood samples were centrifuged at 3500 rpm for 15 min, and the serum was separated into 1.5 mL centrifuge tubes and stored at −20 °C. IL-1, IL-6 and TNF-α were detected by ELISA. (BPRO, Lengton Bioscience Co., Ltd., Shanghai, China).

#### 2.4.7. Lung Tissue Inflammatory Factors

According to the weight/volume ratio of 1:19, the lung tissue was grinded with normal saline, and the supernatant was obtained by 3000 r/min centrifugation for 10 min. According to the instructions of the kit, the expression of IL-1 β, IL-6 and TNF- α in lung tissue was detected. (BPRO, Lengton Bioscience Co., Ltd., Shanghai, China).

### 2.5. Statistical Analysis

Data were expressed as mean ± SEM and analyzed by one-way and two-way ANOVA for single-factor and two-factor designs, respectively, using SPSS (SPSS statistics 20). In the case of the two-way analysis, interactions between treatment factors were also assessed using the SPSS program. The mean differences among different treatments were separated by Duncan’s multiple range tests. A level of *p* < 0.05 was used as the criterion for statistical significance.

## 3. Results

### 3.1. Clinical Signs

**Figure 1:**
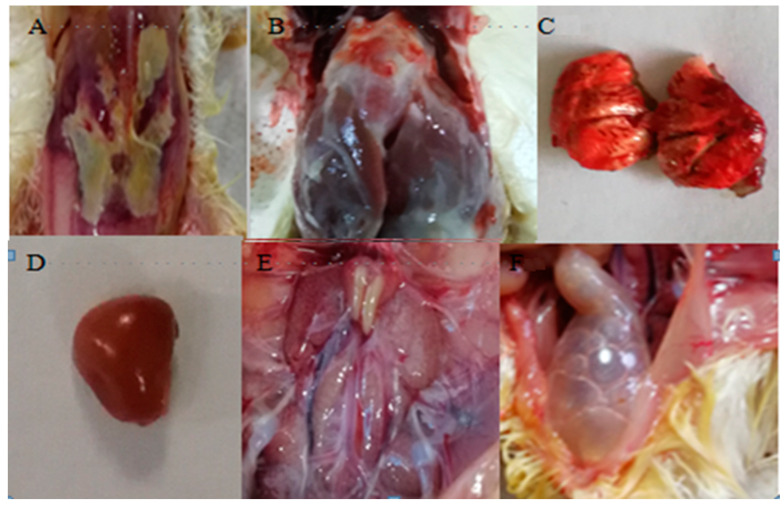
Gross examination of dead chickens. (**A**) subcutaneous fibrinous exudate; (**B**) fibrinous exudate on the heart and liver; (**C**) hemorrhagic necrosis of the lung; (**D**) splenomegaly; (**E**) renal enlargement; (**F**) intestinal distention.

**Figure 2:**
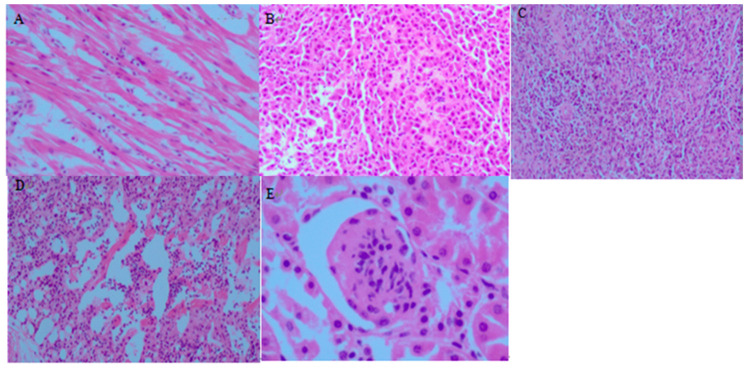
HE pathological section (**A**) myocardial fiber degeneration and gap widening (×100); (**B**) hemolysis of interfibrillar veins in the liver with fatty degeneration (×100); (**C**) unclear structure of splenic vesicles with a large number of lymphocytes visible (×100); (**D**) pulmonary bruising with dilated capillaries in the alveolar wall (×100); (**E**) enlarged gap in the glomerular wall (×400).

Chicks infected with APEC showed typical clinical signs, including depression, anorexia, and inactivity, with a 100% incidence rate. Considering the high mortality rate of chicks (60%), the typical clinical signs and the appearance of autopsy changes, it shows that the model used in our study was successful. Gross examination revealed subcutaneous fibrinous exudates, severe pericarditis, perihepatitis, hemorrhagic necrosis of the lungs, enlarged spleen and kidneys and inflated intestine in dead chickens (Figure 1 and Figure 2).

**Figure 3:**
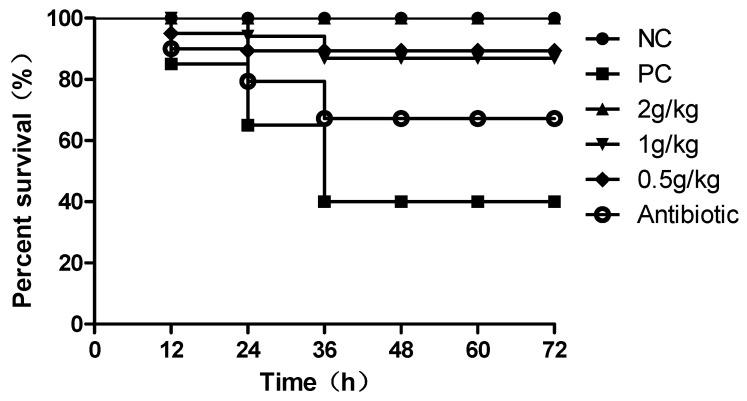
Survival curve. Survival curves depict the survival of *E. coli* infected chickens treated with XCHD.

After treatment, the diet and drinking water returned to normal compared to the PC. The therapeutic effects of each group were as follows: Table 2 showed that the survival rate of each group was significantly higher than that of the Antibiotic group. Survival curves are indicative of these results (Figure 3). Compared to the PC group, chicken mortality was reduced by more than 50% in the XCHD treatment group and 30% in the antibiotic group.

### 3.2. Body Weight

**Figure 4:**
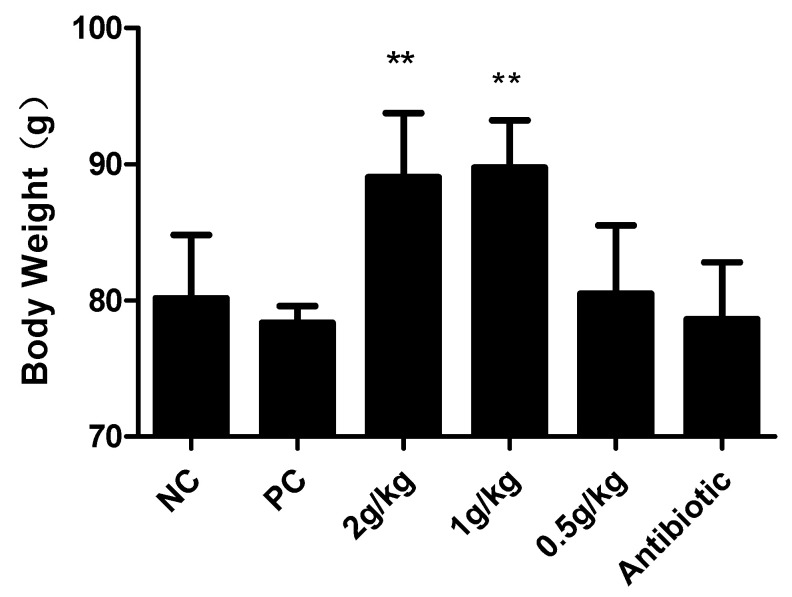
Mean body weight (g) of chickens in different groups at the end of the experiment. Data were presented as mean ± SD (n = 20). ** *p* < 0.01 are significantly different from the NC group.

Mean body weights of different groups are illustrated in Figure 4. At the end of the observation, the mean body weight of 2 g/kg group and 1 g/kg group was higher than that of the PC group. The results demonstrate that XCHD can well restore the weight loss caused by APEC infection as 2 g/kg group and 1 g/kg group increased weight compared to the NC group.

### 3.3. Organ Index

Typical pathological changes of APEC could be seen in the PC group during the treatment, with varying degrees of swelling in the organs, causing an increase in organ index. The examination results were also alleviated after treatment. Organ indices showed a significant reduction in the increase of organ indices caused by APEC in all groups (Table 3).

### 3.4. Determination of Bacterial Load in Organs

**Figure 5:**
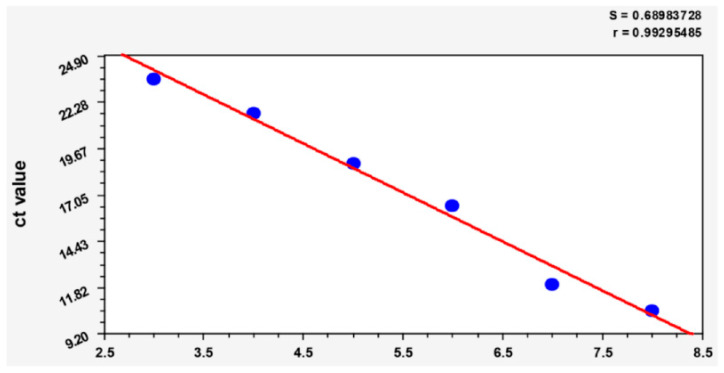
Standard curve of bacterial load in organs.

The DNA extracts of 10-fold dilutions from 10^8^ to 10^2^ cfu/mL were used for qPCR assays to establish the standard curve (Figure 5. A linear relationship between the log10 cfu and the threshold cycle Ct values of (r = 0.99).

Visceral bacterial load in all treatment groups was lower than PC group, the Antibiotic group significantly inhibited the proliferation of APEC in the lung and liver (Table 4)

### 3.5. Blood Biochemical Indicators

**Figure 6:**
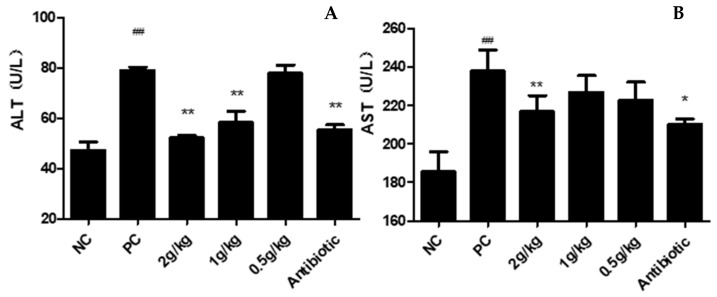
**Effect of XCHD on APEC-induced blood biochemical indicators production.** (**A**) Effects of XCHD on serum ALT activities; (**B**) Effects of XCHD on serum AST activities. Data were presented as mean ± SD (n = 5). ## *p* < 0.01 is significantly different from the NC group; * *p* < 0.05 and ** *p* < 0.01 are significantly different from the PC group.

The biochemical index test showed that the biochemical indexes AST and ALT indexes of the chicks in the PC group after infection were significantly higher than NC group. After XCHD treatment, each administration group can significantly reduce the ALT index of the experimental chicks; both the 2 g/kg dose group and the antibiotic group can significantly reduce the AST and ALT index of the experimental chicks (Figure 6). The results showed that both the XCHD treatment group and the antibiotic group could reduce the liver injury caused by APEC infection.

### 3.6. Serum Inflammatory Factors

**Figure 7:**
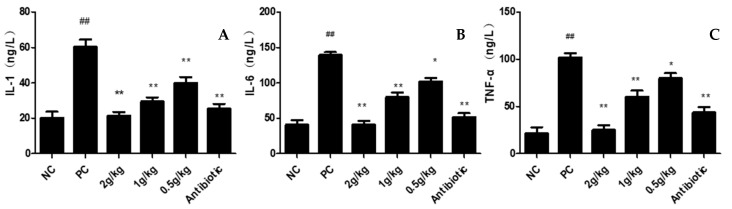
**Effect of XCHD on APEC-induced inflammatory cytokine production**. (**A**) Effects of XCHD on serum IL-1β activities; (**B**) Effects of XCHD on serum IL-6 activities; (**C**) Effects of XCHD on serum TNF-α activities. Data were presented as mean ± SD (n = 5). ## *p* < 0.01 is significantly different from the NC group; * *p* < 0.05 and ** *p* < 0.01 are significantly different from the PC group.

The results showed that the levels of IL-1β, IL-6 and TNF-α in the model group were significantly higher than in the NC group. In addition, serum IL-1, IL-6 and TNF-α secretions were inhibited significantly in all XCHD groups, especially in high doses (Figure 7). The results showed that both the XCHD group and the antibiotic group had good anti-inflammatory effects, proving that XCHD could control systemic inflammation.

### 3.7. Lung Tissue Inflammatory Factors

**Figure 8:**
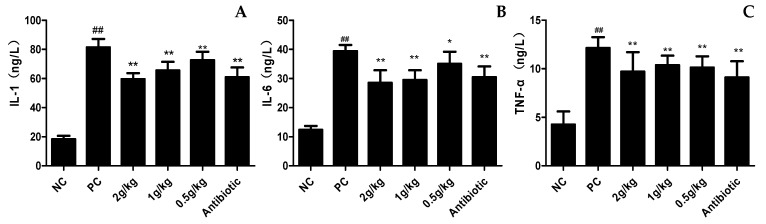
**Effect of XCHD on APEC-induced inflammatory cytokine expression in lung tissue.** (**A**) Effects of XCHD IL-1β activities in lung tissue; (**B**) Effects of XCHD on serum IL-6 activities in lung tissue; (**C**) Effects of XCHD on serum TNF-α activities in lung tissue. Data were presented as mean ± SD (n = 5). ## *p* < 0.01 is significantly different from the NC group; * *p* < 0.05 and ** *p* < 0.01 are significantly different from the PC group.

The results showed that compared with the control group, model group induced the production ofIL-1 β, IL-6 and TNF- α, in lung tissue. The secretion of IL-1, IL-6 and TNF-α in lung tissue was significantly inhibited in all treatment groups, especially in the high dose group. (Figure 8). The results showed that both the XCHD group and the antibiotic group had good anti-pulmonary inflammatory effects, proving that XCHD could control the inflammation of the lung.

## 4. Discussion

APEC associated with colibacillosis results in high morbidity and mortality, and severe economic losses to the poultry industry. APEC is a zoonotic pathogen and can infect humans through contaminated poultry products. Vaccination and antibiotic treatment are currently used to control APEC infections; however, the limited effect of vaccines and the emergence of antibiotic-resistant strains have necessitated the development of novel therapeutics. 

In a previous study, our team had used either laryngeal inoculation or intraperitoneal injection of APEC and found that the mortality rate of chickens in the laryngeal inoculation model was low at about 20–40%, with mild lesions on gross examination, which did not replicate the chicken colibacillosis model well. The mortality rate of chickens in the intraperitoneal injection model was high at about 80%, and the infected chickens died very rapidly due to acute sepsis. Therefore, in the present study we chose pectoral muscle injection of APEC to establish the model of chicken colibacillosis (Table S3).

To address the serious problem of bacterial resistance, researchers are now seeking new alternative antibiotic drugs. There have been many studies showing many new antibiotic alternatives, such as Schisandrin A, small molecule growth inhibitors (GIs) and quorum sensing autoinducer-2 (QS AI-2) inhibitors [25,27,28]. The main mechanisms include regulation of the hepatic-intestinal axis, anti-inflammatory, reduces bacterial load on organs, etc.

In our study the results showed that XCHD was effective in the treatment of chicken colibacillosis, and the healing rate was higher than that of the antibiotic group. There was no significant difference between groups treated with XCHD, although the cure rate was dose related. In addition, the body weight value of birds treated with XCHD was greater than that of the model group, suggesting that XCHD could reduce the negative effects of APEC infection in chickens. At 36 h after infection by APEC, the mortality rates of the model chickens were highest while the mortality rate and body temperature of XCHD-treated chickens were significantly improved compared with the PC group.

We speculate that animal temperatures may be related to the body’s immunity. When the body temperature returns to normal, the body’s resistance is stronger and the survival rate of animals is higher. The APEC infection causes inflammation of chicken organs, with swelling and bleeding, which can be well demonstrated by organ index. However, sometimes the organs do not expand, the weight of the bird decreases and the organ index increases. In this study, however, no weight loss was observed in chickens infected with APEC, and the increase in spleen index was significant. The immune organ indexes are essential in evaluating the speed and state of the immune system [29]. As the largest peripheral immune organ, the spleen can produce an immune response to blood antigen. Therefore, LPS can stimulate lymphocyte overgrowth and induce inflammatory cytokine production on a large scale, leading to splenomegaly [30].

After XCHD treatment, the load of APEC in each organ decreased in different degrees compared with the model. Ceftiofur sodium was selected as an antibiotic and is widely used in veterinary clinics. It has good therapeutic effect on APEC. The pharmacokinetics of ceftiofur sodium, which can be absorbed by subcutaneous injection and distributed to various tissues and organs, showed that the main absorption site was the lung. Our experimental results show that after ceftiofur sodium treatment, the bacterial load in the lungs and liver is significantly reduced, therefore, it is speculated that the liver may also be one of its targets. Cephalosporins reduce levels of inflammation throughout the body by inhibiting damage to the liver by APEC to reduce the mortality rate from APEC infection.

After APEC infection, it breaks through host defense systems, causing tissue damage and host inflammation., By testing the AST and ALT indicators, the results showed that the model group chickens were significantly higher than the control group, which may be due to infection-induced perihepatitis. and the secretion of inflammatory cytokines such as IL-6, and TNF-α also supports this phenomenon. IL-6 is a typical inflammatory factor that is highly expressed when tissue cells are infected with the pathogen. Disease conditions, such as local and systemic infections, septic shock, degenerative arthritis and other autoimmune diseases appear to be regulated by TNF-α and IL-1β. 

As a unique structure in poultry, air sac is an “open” system from nasal cavity to lungs, abdominal organs, bones and related tissues. At the same time, poultry have no diaphragm, and the chest is connected to the abdominal cavity. These special structures determine that poultry are more prone to lung injury [31,32]. Pathogenic factors of lung injury induce systemic infection and lead to systemic inflammatory reaction. In the process of inflammation, a variety of inflammatory cytokines (IL-1, TNF- α, etc.) play an important role in inducing tissue injury. As the target organ of systemic inflammatory response, inflammatory mediators enter the lungs through blood circulation, inducing related inflammatory cells (such as chemotactic monocytes, lymphocytes, polymorphonuclear cells and platelets) to accumulate in the lungs, which in turn causes inflammatory injury. Clinical inhibition of inflammatory cytokines and reduction of inflammatory response has become one of the important methods to reduce lung injury. Some studies have shown that some drugs can effectively suppress these diseases. For instance, some drugs can regulate TNF-α receptor antagonist, IL-6 receptor antagonist and IL-1 receptor antagonist and reverse endotoxin-mediated death. Therefore, we speculate that XCHD can antagonize these three inflammatory cytokines and prevent the death of infected chickens. In order to test this hypothesis, we measured the levels of TNF- α, IL-1 and IL-6 in serum and lung tissue of chickens at the end of the experiment. The results showed that XCHD could significantly reduce the concentrations of IL-1, IL-6 and TNF- α in infected chicks, indicating that XCHD can treat chicken colibacillosis by inhibiting systemic and pulmonary inflammation.

## 5. Conclusions

The results showed that XCHD could improve the survival rate of infected chickens. XCHD can improve the body weight of chickens, reduce the swelling of organs, reduce their bacterial load, reduce liver damage caused by APEC infection and inhibit the secretion of serum and pulmonary inflammatory factors IL-1β, IL-6 and TNF- α. These actions ultimately promote the overall health and growth of the chicken.

## Figures and Tables

**Table 1:** Grouping and treatment of animals.

Group	Number	Treatment
Negative Control (NC)	20	Injected 0.4 mL saline into the pectoral muscle, and then 0.4 mL saline was administered by gavage twice daily for 3 days and followed by 4 days of observation.
Positive Control (PC)	20	Injected 0.4 mL APEC into the chest muscle, and then 0.4 mL saline was administered by gavage twice daily for 3 days and followed by 4 days of observation.
2 g/kg	20	The drug was administered by gavage 3 h after APEC was injected, twice daily for 3 days and followed by 4 days of observation.
1 g/kg	20	The drug was administered by gavage 3 h after APEC was injected, twice daily for 3 days and followed by 4 days of observation.
0.5 g/kg	20	The drug was administered by gavage 3 h after APEC was injected, twice daily for 3 days and followed by 4 days of observation.
Antibiotic	20	*E. coli* was injected and after 3 h, the drug was given once a day, and 0.1 mL/was used continuously for 3 days and followed by 4 days of observation.

**Table 2:** Therapeutic effect of XCHD in chickens colibacillosis.

Group	Number	Morbidity (%)	Mortality (%)	Survival Rate (%)
NC	20	0	0	100
PC	20	100	60	40
2 g/kg	20	100	0	100
1 g/kg	20	100	10	90
0.5 g/kg	20	100	10	90
Antibiotic	20	100	30	70

**Table 3:** Organ index of chickens in each group.

Group	Organ Index					
	Heart	Liver	Spleen	Lung	Kidney	Bursa of Fabricius
NC	8.24 ± 0.94	32.31 ± 2.74	0.79 ± 0.55	9.97 ± 1.46	12.74 ± 1.25	2.08 ± 0.86
PC	20.05 ± 3.77 **	55.61 ± 10.04 **	1.99 ± 0.95 *	9.92 ± 1.81	15.00 ± 8.53 **	1.97 ± 0.72
2 g/kg	9.24 ± 1.36	43.54 ± 7.00	1.36 ± 0.48	7.04 ± 2.36	14.55 ± 1.59	1.53 ± 0.63
1 g/kg	9.63 ± 2.90	40.43 ± 5.18	1.04 ± 0.40	7.49 ± 1.23	15.47 ± 1.53	1.80 ± 0.99
0.5 g/kg	8.89 ± 0.54	37.38 ± 1.37	0.98 ± 0.22	8.65 ± 1.37	14.27 ± 1.03	1.53 ± 0.37
Antibiotic	7.96 ± 1.83	38.90 ± 10.19	1.39 ± 0.73	8.62 ± 1.32	13.76 ± 2.69	1.57 ± 0.89

Data were presented as mean ± SD (n = 5). * *p* < 0.05 and ** *p* < 0.01 are significantly different from the NC group.

**Table 4:** Effect of drugs on visceral bacterial load of chickens in each group.

Organ	Bacterial Load in Organs (lg10 cfu/0.1 g)				
	PC	2 g/kg	1 g/kg	0.5 g/kg	Antibiotic
Heart	3.84 ± 0.98	2.64 ± 0.12	3.21 ± 0.16	3.71 ± 0.11	3.06 ± 0.12
Liver	3.53 ± 0.11	2.63 ± 0.34	2.57 ± 0.36	2.41 ± 0.21	2.35 ± 0.20 **
Spleen	3.31 ± 0.78	2.81 ± 0.56	2.77 ± 0.66	2.71 ± 0.43	3.02 ± 0.08
Lung	3.08 ± 0.19	2.88 ± 0.32	2.80 ± 0.23	3.04 ± 0.07	2.24 ± 0.54 **
Kidney	2.99 ± 0.33	2.64 ± 0.44	2.59 ± 0.56	2.85 ± 0.56	2.93 ± 0.32
Bursa of Fabricius	3.44 ± 0.24	2.62 ± 0.35	2.60 ± 0.55	2.79 ± 0.23	3.41 ± 0.20

Data were presented as mean ± SD (n = 5). ** *p* < 0.01 are significantly different from the NC group.

## Data Availability

The data that support the findings of this study are available from the corresponding author, upon reasonable request.

## Supplementary Materials

The following supporting information can be downloaded at: https://www.mdpi.com/article/10.3390/pathogens12010030/s1, Table S1:Therapeutic effect of XCHD in chickens colibacillosis; Figure S1: Mean body weight (g) of chickens in different experimental groups at the end of the experiment; Table S2: Organ index of chickens in each group; Figure S2: Effect of XCHD on APEC-induced Blood biochemical indicators production; Table S3: Effect of different inoculation doses on the establishment of chicken colibacillosis model; Figure S3: Gross examination of infected chickens; Figure S4: Internal organs of treated and non-treated chickens.

## References

[1] Alonso M.Z., Padola N.L., Parma A.E., Lucchesi P.M. (2011). Enteropathogenic *Escherichia coli* contamination at different stages of the chicken slaughtering process. Poult. Sci..

[2] Kogovšek P., Ambrožič-Avguštin J., Dovč A., Dreo T., Hristov H., Krapež U., Ravnikar M., Slavec B., Lotrič M., Žel J. (2019). Loop-mediated isothermal amplification: Rapid molecular detection of virulence genes associated with avian pathogenic *Escherichia coli* in poultry. Poult. Sci..

[3] Solà-Ginés M., Cameron-Veas K., Badiola I., Dolz R., Majó N., Dahbi G., Viso S., Mora A., Blanco J., Piedra-Carrasco N. (2015). Diversity of Multi-Drug Resistant Avian Pathogenic *Escherichia coli* (APEC) Causing Outbreaks of Colibacillosis in Broilers during 2012 in Spain. PLoS ONE.

[4] Saha O., Hoque M.N., Islam O.K., Rahaman M.M., Sultana M., Hossain M.A. (2020). Multidrug-Resistant Avian Pathogenic *Escherichia coli* Strains and Association of Their Virulence Genes in Bangladesh. Microorganisms.

[5] Lv G. (2010). Diagnosis and control of fowl colibacillosis. Livest. Poult. Ind..

[6] Logue C.M., Wannemuehler Y., Nicholson B.A., Doetkott C., Barbieri N.L., Nolan L.K. (2017). Comparative Analysis of Phylogenetic Assignment of Human and Avian ExPEC and Fecal Commensal *Escherichia coli* Using the (Previous and Revised) Clermont Phylogenetic Typing Methods and its Impact on Avian Pathogenic *Escherichia coli* (APEC) Classification. Front. Microbiol..

[7] Navi B.B., Kasner S.E., Elkind M.S.V., Cushman M., Bang O.Y., DeAngelis L.M. (2021). Cancer and Embolic Stroke of Undetermined Source. Stroke.

[8] da Rosa G., Dazuk V., Alba D.F., Galli G.M., Molosse V., Boiago M.M., Souza C.F., Abbad L.B., Baldissera M.D., Stefani L.M. (2020). Curcumin addition in diet of laying hens under cold stress has antioxidant and antimicrobial effects and improves bird health and egg quality. J. Therm. Biol..

[9] Yesilbag D., Gezen S.S., Biricik H., Meral Y. (2013). Effects of dietary rosemary and oregano volatile oil mixture on quail performance, egg traits and egg oxidative stability. Br. Poult. Sci..

[10] Li P., Wu M., Xiong W., Li J., An Y., Ren J., Xie Y., Xue H., Yan D., Li M. (2020). Saikosaponin-d ameliorates dextran sulfate sodium-induced colitis by suppressing NF-κB activation and modulating the gut microbiota in mice. Int. Immunopharmacol..

[11] Liang N., Yang G.L., Zhang Z., Liu Y., Li J., Liu X., Liang S., Nikolova D., Jakobsen J.C., Gluud C. (2019). Xiao Chai Hu Tang, a herbal medicine, for chronic hepatitis B. Cochrane Database Syst. Rev..

[12] Wang Y.X., Du Y., Liu X.F., Yang F.X., Wu X., Tan L., Lu Y.H., Zhang J.W., Zhou F., Wang G.J. (2019). A hepatoprotection study of Radix Bupleuri on acetaminophen-induced liver injury based on CYP450 inhibition. Chin. J. Nat. Med..

[13] Qin X.K., Li P., Han M., Liu J.P. (2010). Xiaochaihu Tang for treatment of chronic hepatitis B: A systematic review of randomized trials. Zhong Xi Yi Jie He Xue Bao.

[14] Li J., Hu R., Xu S., Li Y., Qin Y., Wu Q., Xiao Z. (2017). Xiaochaihutang attenuates liver fibrosis by activation of Nrf2 pathway in rats. Biomed. Pharmacother..

[15] Jeon W.Y., Shin H.K., Shin I.S., Kim S.K., Lee M.Y. (2015). Soshiho-tang water extract inhibits ovalbumin-induced airway inflammation via the regulation of heme oxygenase-1. BMC Complement. Altern. Med..

[16] Chen Q.Y., Wang C.Q., Yang Z.W., Tang Q., Tan H.R., Wang X., Cai S.Q. (2017). Differences in anti-inflammatory effects between two specifications of *Scutellariae* Radix in LPS-induced macrophages in vitro. Chin. J. Nat. Med..

[17] Kim D.H., Hossain M.A., Kang Y.J., Jang J.Y., Lee Y.J., Im E., Yoon J.H., Kim H.S., Chung H.Y., Kim N.D. (2013). Baicalein, an active component of *Scutellaria baicalensis* Georgi, induces apoptosis in human colon cancer cells and prevents AOM/DSS-induced colon cancer in mice. Int. J. Oncol..

[18] Kim M.H., Lee H., Choi Y.Y., Lee D.H., Yang W.M. (2018). *Scutellaria baicalensis* ameliorates the destruction of periodontal ligament via inhibition of inflammatory cytokine expression. J. Chin. Med. Assoc. JCMA.

[19] Shen J., Li P., Liu S., Liu Q., Li Y., Sun Y., He C., Xiao P. (2021). Traditional uses, ten-years research progress on phytochemistry and pharmacology, and clinical studies of the genus *Scutellaria*. J. Ethnopharmacol..

[20] Jung S., Park J., Park J., Jo H., Seo C.S., Jeon W.Y., Lee M.Y., Kwon B.I. (2020). *Sojadodamgangki-tang* attenuates allergic lung inflammation by inhibiting T helper 2 cells and Augmenting alveolar macrophages. J. Ethnopharmacol..

[21] Hsu B.Y., Kuo Y.C., Chen B.H. (2014). Polysaccharide Isolated from *Zizyphus jujuba* (Hóng Zǎo) Inhibits Interleukin-2 Production in Jurkat T Cells. J. Tradit. Complement. Med..

[22] Wang D., Zhao Y., Jiao Y., Yu L., Yang S., Yang X. (2012). Antioxidative and hepatoprotective effects of the polysaccharides from *Zizyphus jujube* cv. Shaanbeitanzao. Carbohydr. Polym..

[23] Jing M., Munyaka P.M., Tactacan G.B., Rodriguez-Lecompte J.C., O K., House J.D. (2014). Performance, serum biochemical responses, and gene expression of intestinal folate transporters of young and older laying hens in response to dietary folic acid supplementation and challenge with *Escherichia coli* lipopolysaccharide. Poult. Sci..

[24] Cesta M.F. (2006). Normal structure, function, and histology of the spleen. Toxicol. Pathol..

[25] Bao J., Zhang Y., Zhang L., Gong X., Shi W., Liu L., Wang X. (2021). Therapeutic effect of Schisandrin A on avian colibacillosis through gut-liver axis. Poult Sci..

[26] Lv Z., Fan H., Song B., Li G., Liu D., Guo Y. (2019). Supplementing Genistein for Breeder Hens Alters the Fatty Acid Metabolism and Growth Performance of Offsprings by Epigenetic Modification. Oxid. Med. Cell. Longev..

[27] Helmy Y.A., Kathayat D., Deblais L., Srivastava V., Closs G., Tokarski R.J., Ayinde O., Fuchs J.R., Rajashekara G. (2022). Evaluation of Novel Quorum Sensing Inhibitors Targeting Auto-Inducer 2 (AI-2) for the Control of Avian Pathogenic *Escherichia coli* Infections in Chickens. Microbiol. Spectr..

[28] Kathayat D., Helmy Y.A., Deblais L., Srivastava V., Closs Jr G., Khupse R., Rajashekara G. (2021). Novel Small Molecule Growth Inhibitor Affecting Bacterial Outer Membrane Reduces Extraintestinal Pathogenic *Escherichia coli* (ExPEC) Infection in Avian Model. Microbiol. Spectr..

[29] Luppi P., Licata A., Haluszczak C., Rudert W.A., Trucco G., McGowan F.X., Finegold D., Boyle G.J., Trucco M. (2001). Analysis of TCR Vbeta repertoire and cytokine gene expression in patients with idiopathic dilated cardiomyopathy. J. Autoimmun..

[30] Diel R., Hauer B., Loddenkemper R., Manger B., Krüger K. (2009). Recommendations for tuberculosis screening before initiation of TNF-alpha-inhibitor treatment in rheumatic diseases. Pneumologie.

[31] Maina J.N. (2003). Developmental dynamics of the bronchial (airway) and air sac systems of the avian respiratory system from day 3 to day 26 of life: A scanning electron microscopic study of the domestic fowl, *Gallus gallus* variant *domesticus*. Anat. Embryol..

[32] Letsiou E., Sammani S., Wang H., Belvitch P., Dudek S.M. (2017). Parkin regulates lipopolysaccharide-induced proinflammatory responses in acute lung injury. Transl. Res. J. Lab. Clin. Med..

