# Supplementary material for: Xiaochaihu Decoction Treatment of Chicken Colibacillosis by Improving Pulmonary Inflammation and Systemic Inflammation
Source: Pathogens. 2022 Dec 25;12(1):30. doi: 10.3390/pathogens12010030 (PMC9862048; doi:10.3390/pathogens12010030)

Supplement materials

1. Screening of effective drug doses for XCHD

The chicken were reared to 12 days of age and randomly divided into 7 groups: NCgroup, PC group, 4 g/kg, 2 g/kg, 1 g/kg, 0.5 g/kg, 0.25 g/kg, and 20 chicks in each group. The chicks were randomly divided into 7 groups, namely blank group, model group, 4 g/kg, 2 g/kg, 1 g/kg, 0.5 g/kg, 0.25 g/kg dose group, 20 chicks in each group.

Except for the NC group, chickens in each group were injected with 0.4 mL of 10^8 cfu/mL of APEC solution into the pectoral muscle, and clinical symptoms were observed 3 h after the infection. The drug was administered by gavage 3 hours after APEC injected, twice daily for 3 days, followed by 4 days of observation.

Observations included mortality and survival rates, changes in body weight, changes in organ indices, and changes in biochemical parameters of chickens.

Table S1 Therapeutic effect of XCHD in chickens colibacillosis.

Group	number	Morbidity (%)	Mortality (%)	Survival rate (%)
NC	20	0	0	100
PC	20	100	55	45
4g/kg	20	100	0	100
2g/kg	20	100	0	100
1g/kg	20	100	10	90
0.5g/kg	20	100	10	90
0.25g/kg	20	100	10	80

Figure S1. Mean body weight (g) of chickens in different experimental groups at the end of the experiment.

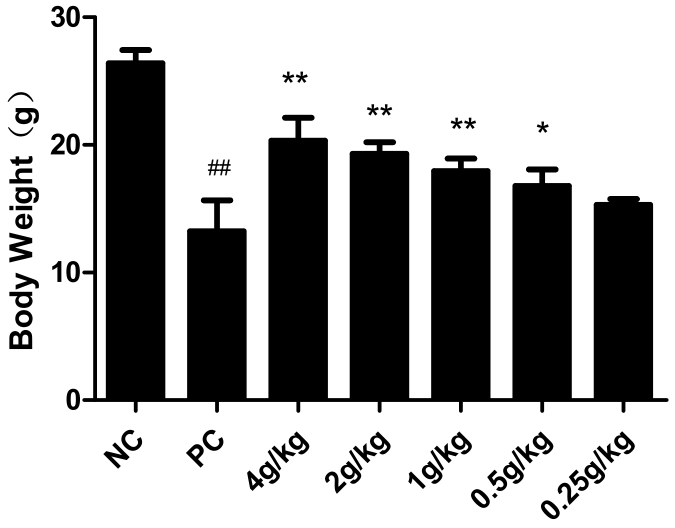

Data were presented as mean ± SD (n = 20). *p < 0.05 and **p < 0.01 are significantly different from the NC group.

At the end of the observation, the mean body weight of 4g/kg, 2 g/kg group, 1 g/kg and 0.5g/kg group was higher than that of the PC group.

Table S2 Organ index of chickens in each group

Group	Organ index					
	Heart	Liver	Spleen	Lung	Kidney	Bursa of Fabricius
NC	6.46±0.90	30.46±4.32	0.93±0.18	7.35±1.19	10.01±0.81	2.76±0.68
PC	9.03±1.23**	46.06±13.92**	1.71±0.48**	9.61±2.35	17.20±3.11**	1.57±0.84
4g/kg	7.54±0.75	26.87±3.13	1.08±0.22	7.78±2.85	11.12±1.33	1.88±0.79
2g/kg	7.57±0.77	28.92±2.09	1.35±0.34	7.83±2.05	12.20±1.14	2.28±0.45
1g/kg	6.96±0.70	32.39±7.94	1.76±0.49**	9.50±2.90	12.81±2.98	2.22±0.45
0.5g/kg	8.28±0.99**	34.75±5.94	1.38±0.32	8.16±3.70	14.58±2.25**	2.06±1.10
0.25g/kg	9.46±2.07**	36.43±4.87	1.75±0.66**	9.55±2.10	14.18±2.73**	2.21±0.87

Data were presented as mean ± SD (n = 5). *p < 0.05 and **p < 0.01 are significantly different from the NC group.

The results of organ index measurement showed that 4g/kg, 2g/kg and 1g/kg groups could reduce the increase of organ index caused by APEC. All the dose groups could reduce the elevated organ index caused by APEC.

Figure S2 Effect of XCHD on APEC-induced Blood biochemical indicators production.

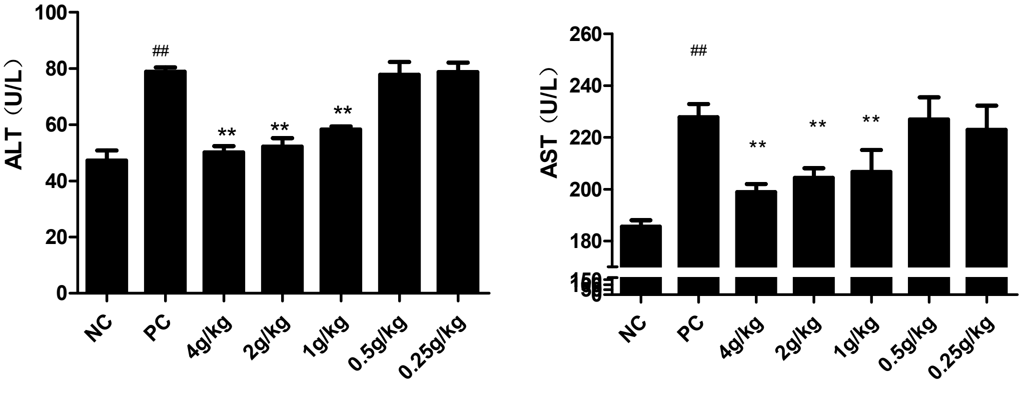

Data were presented as mean ± SD (n = 5). ##p < 0.01 is significantly different from the NC group; *p < 0.05 and **p < 0.01 are significantly different from the PC group.

The biochemical index test showed that the biochemical indexes AST and ALT indexes of the chicks in the PC group after infection were significantly higher than NC group. After XCHD treatment, 4g/kg, 2g/kg and 1g/kg group can significantly reduce the ALT and AST index of the experimental chicks.

Based on these results and the actual XCHD cost, the final choice in this study was 2g/kg, 1g/kg and 0.5g/kg.

2. Effect of different inoculation routes on artificially induced chicken colibacillosis

In total, 60 one-day old male hyline brown chickens (average BW = 50 g) were obtained from Changchun Academy of Agricultural Sciences. They were in specked upon arrival to ensure all chicks were free from any deformity and early signs of disease.

Make the concentration of APEC solution at 10^8 cfu/mL., injection methods include laryngeal, ventral and pectoral injection, each group was inoculated with 0.4mL.

Table S3 Effect of different inoculation doses on the establishment of chicken colibacillosis model

**Group**	**number**	**Morbidity (%)**	**Mortality (%)**	**Survival rate (%)**
Laryngeal	20	50	20	80
Ventral	20	100	80	20
Pectoral	20	100	60	40

Most of the chickens inoculated with larynx were found to die around 48h-72h, and the lesions were not obvious on autopsy. Figure 3a.

Most of the chickens injected intraperitoneally died acutely, the majority within 24h and some within 48h. There were also no obvious changes on autopsy, and occasionally subcutaneous exudation from the abdomen was seen Figure 3b.

Pectorally injected chickens died mostly within 72 h. The examination revealed exudation and hepatomegaly or pericardial fibrinous exudation Figure 3 c.

Figure S3 Gross examination of infected chickens.

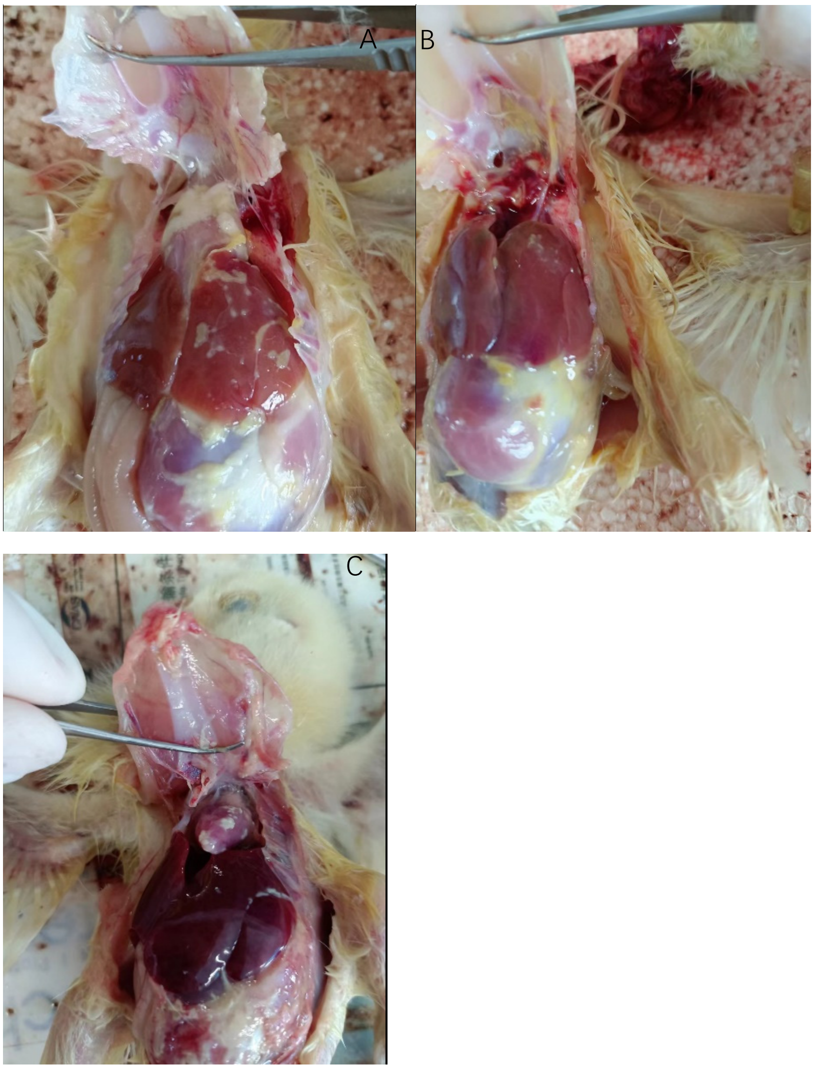

.

Internal organs of treated and non-treated chickens.

Figure S4. A:NC group;　B:PC group；C:2g/kg treatment group；D：1g/kg treatment group；

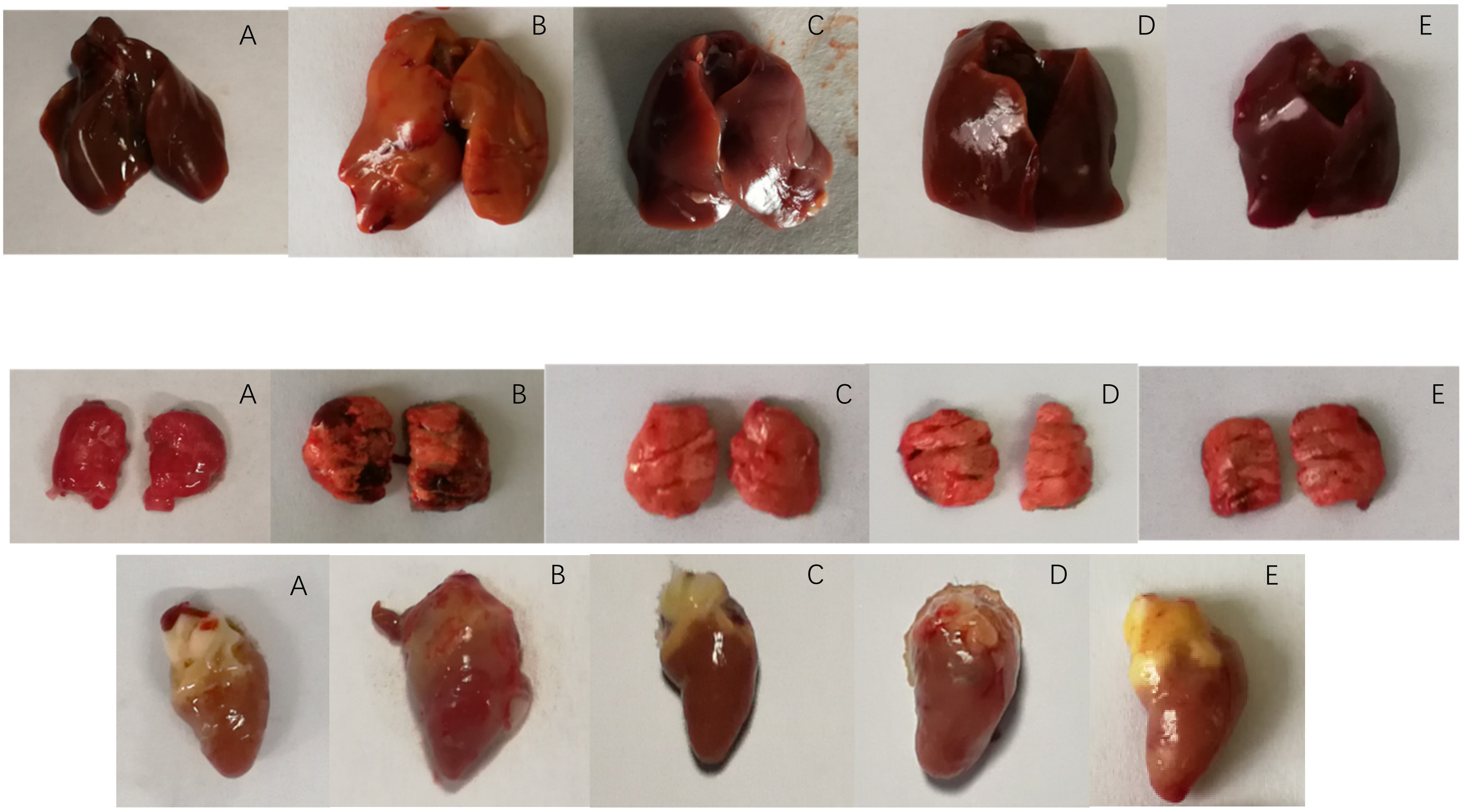

Supplement: Supplementary file 1 [file pathogens-12-00030-s001.zip › pathogens-2053922-supplementary.pdf]
